# Current Controversies in Classification, Management, and Prevention of Bisphosphonate-Related Osteonecrosis of the Jaw

**DOI:** 10.1155/2014/565743

**Published:** 2014-12-21

**Authors:** Giuliano Ascani, Giuseppina Campisi, Luis Manuel Junquera Gutierrez

**Affiliations:** ^1^Department of Maxillofacial Surgery, Spirito Santo Hospital, 65124 Pescara, Italy; ^2^Department of Surgical, Oncological and Oral Sciences, University of Palermo, 90128 Palermo, Italy; ^3^Department of Oral and Maxillofacial Surgery, Central University Hospital of Asturias, University of Oviedo, 33006 Oviedo, Spain

Bisphosphonate-related osteonecrosis of the jaw (BRONJ) is a serious complication associated with oral and intravenous bisphosphonate therapy that adversely affects the quality of life, producing significant morbidity.

Since the first description of bone necrosis in patients receiving bisphosphonate therapy in 2003 [1], hundreds of studies were published about this topic and various national and international medical societies have published protocols and guidelines. Nevertheless, there are still many controversies regarding the classification, management, and prevention of BRONJ.

Even the definition of BRONJ is still debated and changed with the progress of knowledge and experience. According to the original definition of the AAOMS (American Association of Oral and Maxillofacial Surgery) [2, 3] “*Patients may be considered to have BRONJ if all of the following three characteristics are present: (1) Current or previous treatment with a bisphosphonate; (2) Exposed bone in the maxillofacial region that has persisted for more than eight weeks; and (3) No history of radiation therapy to the jaws*.”

Following recognition of the nonexposed BRONJ clinical variant, it became clear that the presence of exposed necrotic bone in the oral cavity is just one of the possible clinical manifestations of BRONJ and is not found in all BRONJ patients. In 2012 the SICMF (Italian Society for Maxillofacial Surgery) and the SIPMO (Italian Society of Oral Pathology and Medicine) proposed a new definition [4]:* “Bisphosphonate related osteonecrosis of the jaw (BRONJ) is an adverse drug reaction described as the progressive destruction and death of bone that affects the mandible or maxilla of patients exposed to the treatment with nitrogen-containing bisphosphonates, in the absence of a previous radiation treatment.”* Recently, this definition was robustly supported by a cross-sectional study on a large population of European patients with exposed and non-exposed bisphosphonate-associated ONJ; where, according to the traditional definition, only 76% of ONJ were diagnosed, and diagnosis in the remaining 24% could not be adjudicated, as they had several abnormal features relating to the jaws but no visible necrotic bone. [5] In parallel, it was demonstrated, in a large multicentre retrospective study, that the severity of ONJ (i.e. the extent of bony disease) as main guide to its management, can be correctly identified if measured by computed tomography (CT), more accurately than by clinical inspection and radiography as proposed by several staging systems, including the widely-used American Association of Oral and Maxillofacial Surgeons (AAOMS) system [6].

Very recently the AAOMS recommends changing the nomenclature of BRONJ [7]; the AAOMS favors the term* medication-related osteonecrosis of the jaw (MRONJ)*. The change is justified to accommodate the growing number of osteonecrosis cases involving the maxilla and mandible associated with other antiresorptive (denosumab) and antiangiogenic therapies.

The interesting and scientifically significant manuscripts selected for publication in this special issue include review articles, clinical studies, and research articles, which represent an important contribution to analyze and try to solve current controversies in classification, management, and prevention of BRONJ.

In the review article “*Bisphosphonate-related osteonecrosis of the jaw: a review of the literature*” the authors offer a perspective on how dentists should manage patients on BPs, to show the benefits of accurately diagnosing BRONJ and to present diagnostic aids and treatments strategies for the condition.

The role of infection in the etiology of bisphosphonate-related osteonecrosis of the jaw (BRONJ) is poorly understood.

In the review article* “Is bisphosphonate-related osteonecrosis of the jaw an infection? A histological and microbiological ten-year summary”* the authors present a systematic review of BRONJ histology and microbiology (including demographics, immunocompromised associations, clinical signs and symptoms, disease severity, antibiotic and surgical treatments, and recovery status) validating that infection should still be considered a prime component in the multifactorial disease.

The review article* “Bisphosphonate associated osteonecrosis of the jaw: an update on pathophysiology, risk factors, and treatment”* is a narrative review of the literature; its aims are to elaborate on the pathological mechanisms behind the condition and also to gather an update on incidence, risk factors, and treatment of bisphosphonate associated osteonecrosis of the jaw.

The review article* “Imaging findings of bisphosphonate-related osteonecrosis of the jaws: a critical review of the quantitative studies”* offers a critical review of published information on the imaging strategies used for diagnosing bisphosphonate associated osteonecrosis of the jaw in patients taking intravenous bisphosphonates, pointing at the different methodologies and results of existing literature.

The existing BRONJ staging systems are numerous, but not one is surgical oriented.

In the clinical study “*New dimensional staging of bisphosphonate-related osteonecrosis of the jaw allowing a guided surgical treatment protocol: long-term follow-up of 266 lesions in neoplastic and osteoporotic patients from the University of Bari*” a new dimensional stage classification, guiding the surgical treatment of BRONJ patients, is proposed, and the success rate of this new management is evaluated.

The most debated topic about BRONJ is therapy and the most adequate procedure is far from being standardized. Several approaches have been evaluated for the treatment of patients who developed BRONJ and many management strategies have been proposed. Nevertheless, it seems that taking preventative measures is the most effective way to face BRONJ.

In the clinical study “*Platelet rich plasma in the treatment of bisphosphonate-related osteonecrosis of the jaw: personal experience and review of the literature*” the authors considered a group of patients affected by BRONJ with nonsurgical therapy, surgical therapy, and surgical therapy with platelet rich plasma (PRP) gel to evaluate its therapeutic effect in promoting BRONJ wounds healing.

In the clinical study* “Conservative treatment of bisphosphonate-related osteonecrosis of the jaw in multiple myeloma patients” *the authors report a retrospective review of all their MM patients who were treated with bisphosphonates and developed BRONJ and discuss management issues.

The aim of the clinical study* “Risk assessment of BRONJ in oncologic patients treated with bisphosphonates: follow-up to 18 months”* is to monitor the BRONJ level of risk in patients with cancer, according to a preventive clinical protocol, which is firstly aimed at reducing risk factors such as the periodontal infections.

In the research article* “The “CROMa” project: a care pathway for clinical management of patients with bisphosphonate exposure” *the authors describe the activity of “CROMa” (Coordination of Research on Osteonecrosis of the Jaws) project of “Sapienza” University of Rome evaluating the risk variables of patients with past, present, or planned BP exposure, treated with periodontics, oral surgery, and operative dentistry procedures in order to treat or prevent BRONJ.

We sincerely hope that the readers can enjoy this special issue and improve their knowledge about BRONJ; we wish that the articles published will encourage further research on classification, management, and prevention of BRONJ.
